# Cost-effectiveness of three screening strategies for atrial fibrillation in Sri Lanka: a decision-tree modelling analysis using community-based prevalence data

**DOI:** 10.1136/bmjgh-2025-019592

**Published:** 2026-03-13

**Authors:** Shribavan Kanesamoorthy, Zainab Abdali, Tiffany E Gooden, Sheron Antony Vethanayagam, Powsiga Uruthirakumar, Chamira Kodippily, Balachandran Kumarendran, Neil Thomas, Krishnarajah Nirantharakumar, Gregory Y H Lip, Mahesan Guruparan, Rashan Haniffa, Surenthirakumaran Rajendra, Abi Beane, Kumaran Subaschandren, Sue Jowett, on behalf of the NIHR Global Health Research Group on Atrial Fibrillation Management

**Affiliations:** 1 Department of Community and Family Medicine, University of Jaffna, Jaffna, Sri Lanka; 2 Department of Applied Health Sciences, University of Birmingham, Birmingham, UK; 3 National Intensive Care Surveillance-Mahidol Oxford Tropical Medicine Research Unit, Colombo, Sri Lanka; 4 Liverpool Centre for Cardiovascular Science, University of Liverpool, Liverpool, UK; 5 Handy Memorial Cardiology Unit, Jaffna Teaching Hospital, Jaffna, Sri Lanka; 6 Centre for Inflammation Research, The University of Edinburgh, Edinburgh, UK

**Keywords:** Screening, Health economics, Cardiovascular disease, Community-based survey

## Abstract

**Introduction:**

Early diagnosis and treatment of atrial fibrillation (AF) are crucial to reduce AF-related complications and associated healthcare costs. In low-resource settings, digital health technologies could help achieve this; however, costs of different screening strategies are key for policy change.

**Methods:**

This decision-tree model representing the Sri Lankan public health system perspective used prevalence data from a community-based cross-sectional study of 10 000 individuals aged ≥50 years in Northern Province, Sri Lanka. Participants were screened for AF using AliveCor, a handheld single-lead ECG device. Three screening strategies (systematic, opportunistic and targeted) were compared against each other. The incremental cost-effectiveness ratio (ICER) is presented, representing the incremental total aggregated cost between screening strategies divided by the incremental number of new detected AF cases to generate a cost per additional new AF cases detected for a 1-year time horizon.

**Results:**

Systematic screening detected 48 new AF cases, and the targeted screening detected 47. Systematic screening was more expensive (Sri Lankan rupees (Rs) 698 422; US$2123) for 10 000 screened individuals compared with targeted screening (Rs 492 002; US$1496) for 7780 screened individuals. Opportunistic screening was the cheapest strategy (Rs 360 617; US$1096) for screening 6556 individuals; however, only 30 new AF cases were identified. The ICER of targeted screening was lower compared with opportunistic screening (Rs 7729; US$23 per additional detected AF case) whereas the ICER of systematic screening compared with opportunistic screening was higher at Rs 18 767 (US$57) per detected AF case. When the systematic screening strategy was compared with targeted screening, the cost per additional detected AF case increased to Rs 206 420 ($628).

**Conclusion:**

Targeted screening with AliveCor was the most cost-effective strategy. Systematic screening, while having similar effectiveness, was not cost-effective due to the high additional costs to detect just one further case. These findings support integrating targeted screening into Sri Lanka’s primary care pathways.

**WHAT IS ALREADY KNOWN ON THIS TOPIC**

- Awareness, detection and management of atrial fibrillation (AF) is suboptimal in Sri Lanka, leading to excess morbidity and mortality.
- Early detection of AF is critical for timely life-saving medication. One way to achieve this is with digital health technologies.

**WHAT THIS STUDY ADDS**

- This study provides evidence that a targeted screening strategy for AF is the most cost-effective strategy to use in Sri Lanka using a handheld single-lead mobile-based ECG.

**HOW THIS STUDY MIGHT AFFECT RESEARCH, PRACTICE OR POLICY**

- As the burden of AF increases, these findings should guide policy in developing an AF screening plan in primary care to reduce costly and debilitating AF-related complications such as stroke.

## Background

 Strokes caused by atrial fibrillation (AF) are associated with high morbidity and mortality,^1^ creating an increased burden on the healthcare system (cost and resources) and patients and their families (costs and time). Treatment with novel oral anticoagulants or vitamin K-based anticoagulants such as warfarin can reduce stroke incidence by 64%^2 3^; thus, early diagnosis is vital for preventing debilitating morbidity and premature death. However, AF often presents asymptomatically or with mild symptoms, and awareness of AF is poor among many individuals within the general population,^4^ leading to underdiagnosis and preventable strokes. This is especially true in low- and middle-income countries (LMICs) where AF prevalence is expected to increase due to an ageing population and an increase in risk factors such as hypertension, diabetes and heart disease.^5^ Though, with an improved awareness of non-communicable diseases and development of primary care in such settings, AF screening and subsequent AF diagnoses may increase.^6^

33% of people with AF are asymptomatic.^7^ As a response, international guidelines recommend screening for AF in the elderly population (age ≥65 years) and this has been implemented in many high-income countries.^8^,^10^ Routine AF screening is not common practice in many LMICs due to underdeveloped and under-resourced primary care facilities and a lack of training, policy and national guidelines.^11^ A 12-lead ECG is the gold standard for confirming a diagnosis of AF; though, many primary care settings in LMICs, including Sri Lanka, lack access to 12-lead ECG machines.^12^ Our previous research in Sri Lanka identified such inefficiencies and barriers with the healthcare system that resulted in people receiving an AF diagnosis only after experiencing life-threatening complications, with a further barrier identified for early diagnosis related to healthcare seeking behaviours.^13^

One way to overcome these barriers for early AF diagnosis is using phone-based/smartphone handheld single-lead ECG devices that can detect AF and be conducted by the public themselves or by a trained non-clinical healthcare professional.^14^ A single-lead ECG can be used to detect AF in community settings or when a patient presents to primary care with an irregular pulse (with or without the signs or symptoms of AF). Thus, time can be saved to initiate treatment and subsequently reduce the risk of strokes and other cardiovascular complications and improve the quality of life for patients with AF.^15^ Using single-lead ECG devices in the community or in the home can reduce visits to larger and busier healthcare facilities and reduce costs for both healthcare systems and patients.^14^ Evidence from high-income countries suggests that single-lead ECGs are likely to be a cost-effective solution, especially if a longer wait for a 12-lead ECG is necessary and if the 12-lead ECG is performed in secondary or tertiary settings, which is usually the case in LMICs.^16 17^

Sri Lanka (a lower-middle income country)^18^ has faced a multitude of difficulties in delivering and managing the country’s healthcare needs. The country’s 30-year civil war ended in 2009; the COVID-19 pandemic adversely impacted both healthcare infrastructure and resources^19^; and the current economic crisis adds further challenges to ensuring healthcare can be provided effectively and efficiently to all who need it.^20^ Sri Lanka has universal healthcare,^21^ providing free medical appointments, medications and medical procedures to all citizens. As the Sri Lankan healthcare system continues to develop and overcome adversities, comparing the financial and social impact of healthcare interventions can aid the Sri Lankan government in choosing the most cost-effective intervention to invest in.

No evidence exists in Sri Lanka, nor any other LMIC, on the costs of different AF screening strategies using single-lead ECGs. Therefore, we conducted the first health economic analysis of AF screening in Sri Lanka, with the aim to estimate and compare the potential costs of detecting AF with a single-lead ECG using three screening strategies: systematic (community-based), opportunistic and targeted screening. Additionally, the analysis sought to estimate the potential number of stroke cases prevented and the cost of managing strokes for the different screening strategies.

## Methods

### Study design

This decision-tree model used prevalence data from a community-based cross-sectional study of 10 000 individuals ≥50 years in Northern Province, Sri Lanka (manuscript under review). Screening outcomes (new AF cases detected) and participant characteristics were derived from this larger study. The methods of the cross-sectional study are explained in detail elsewhere.^22^ In brief, a multistage cluster sampling approach was used. Individuals with a terminal illness, in need of immediate hospital admission or currently an inpatient in a hospital setting, were excluded. Each cluster consisted of 20 households, with one participant per household. Data on demographics, socio-economic status, risk factors, comorbidities and visits to healthcare settings were collected from all participants through a phone-based questionnaire, administered by trained data collectors with medical backgrounds who underwent 2 weeks of intensive training.^22^ Data were collected via a community-based household survey and systematic screening of AF between June 2020 and March 2023 from all five districts in the Northern Province of Sri Lanka: Jaffna, Kilinochchi, Mannar, Mullaitivu and Vavuniya. Screening for AF was performed using AliveCor, a mobile handheld single-lead ECG device, which records heart rhythm through fingertip electrodes and produces a single-lead (lead 1) ECG. The results were recorded through the AliveCor smartphone application that uses an automated algorithm to interpret the cardiac rhythm and identify AF. AliveCor is approved by the Food and Drug Administration and has been validated^23^ in many settings.

Using data from the cross-sectional study, we compared the cost-effectiveness of systematic, targeted and opportunistic case-finding strategies over a 1-year time horizon. The following data from the cross-sectional study were used to inform the sample for each case finding strategy: sociodemographic data, current and past physical and mental health conditions, well-being and lifestyle behaviours (eg, smoking, diet, physical exercise) and healthcare utilisation.^22^

### Economic evaluation

The cost-effectiveness analysis was performed in the form of a decision analytical model (DAM) using a decision tree, representing the Sri Lankan public health system perspective. DAMs are used to synthesise clinical and economic evidence to assess the relative cost-effectiveness of interventions to inform healthcare resource allocation decisions.^24^

Three case finding strategies (systematic, opportunistic and targeted) were compared against each other over a 1-year time horizon. The outcome of the analysis was the incremental cost-effectiveness ratio (ICER) which represents the incremental total aggregated cost between screening strategies divided by the incremental number of new detected AF cases to generate a cost per additional new AF cases detected.

### Model structure

**Figure 1:**
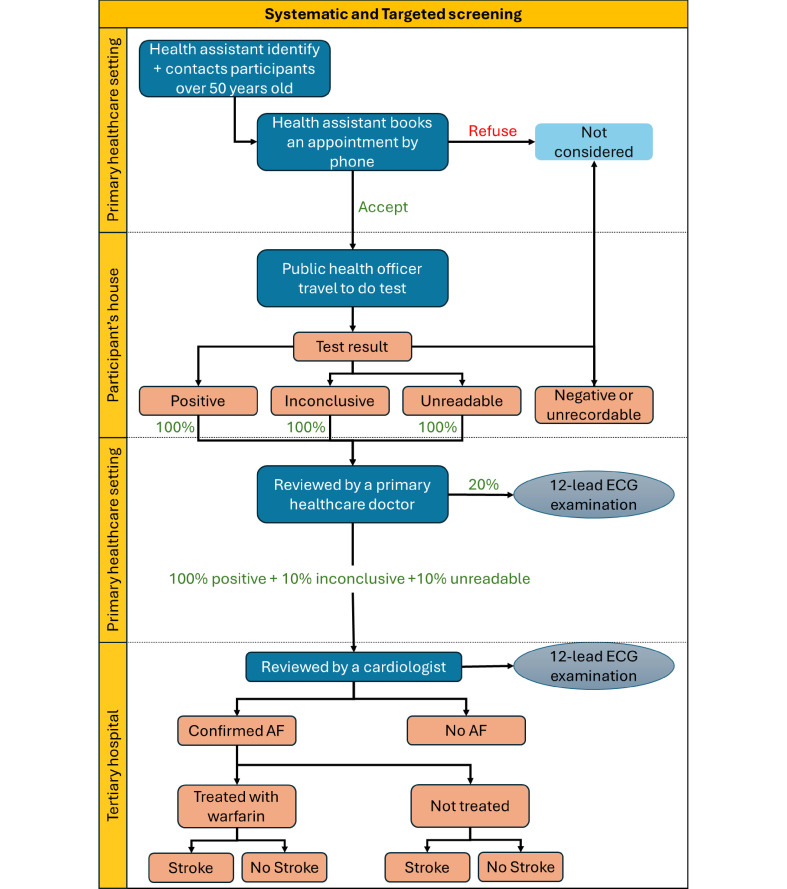
Diagnostic and post-diagnostic pathway—systematic and targeted screening strategies. AF, atrial fibrillation.

The decision tree model reflects the care pathways for AF in a real-world setting for non-institutionalised permanent residents in Sri Lanka. The care pathways were conceptualised using information from the cross-sectional study in addition to discussions with clinical experts from Sri Lanka experienced in the field of cardiology. The systematic population-based case-finding strategy was based directly on the data from the cross-sectional study. In the care pathway for systematic screening of the model (figure 1), individuals aged 50 years or above are contacted via phone from dedicated healthcare assistants from primary healthcare units and are invited to visit the nearest primary healthcare facility. During the healthcare visit, an AliveCor recording is performed by a public health officer and individuals with a positive, inconclusive or unreadable result are initially reviewed by the primary healthcare doctor and a 12-lead ECG is performed when available (estimated at 20% of primary care units). All individuals with a positive AliveCor result and 10% of those with an inconclusive or unreadable AliveCor result are estimated for a referral and a scheduled appointment with a cardiologist at a tertiary care hospital. The cardiologist then reviews the AliveCor results and orders a 12-lead ECG for all individuals referred. The same care pathway was used for the targeted screening of the model (figure 1); however, only individuals aged 60 years or older with one of the following risk factors are considered for an invitation to be screened: a diagnosis of hypertension, diabetes and/or a cardiac disease (ischaemic heart disease, transient ischaemic attack, stroke or peripheral vascular disease). Data collected on health conditions and lifestyle behaviours were used to determine who would be included in a targeted screening strategy.

**Figure 2:**
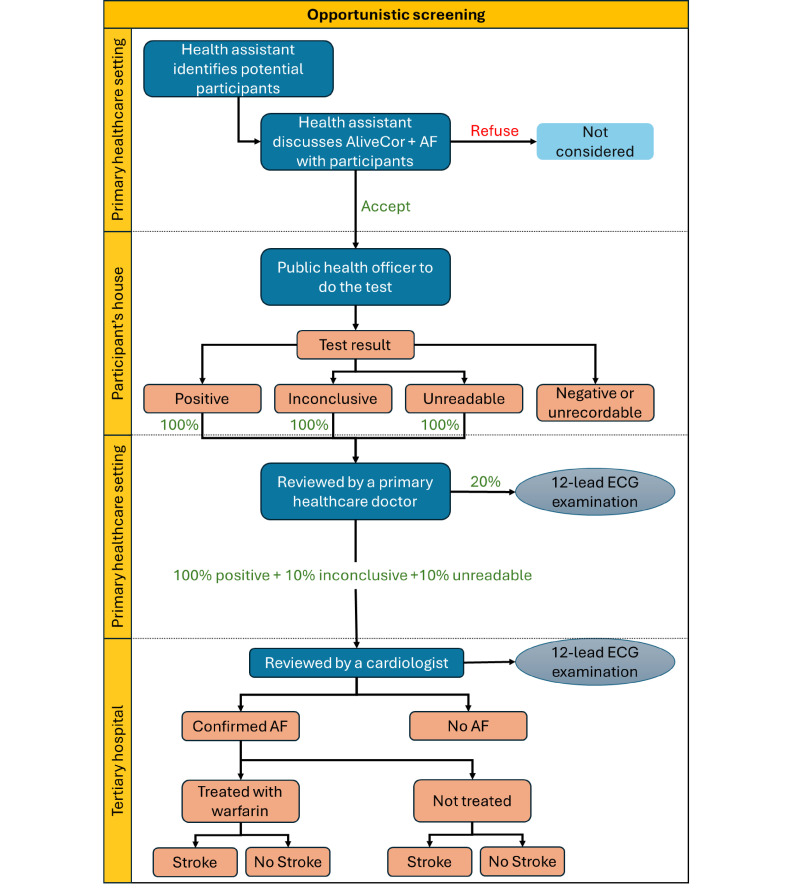
Diagnostic and post-diagnostic pathway—opportunistic screening strategy. AF, atrial fibrillation.

In the opportunistic screening of the model (figure 2), the care pathway was assumed to only involve individuals that made any visit to a primary healthcare unit, divisional hospital or outpatient department within the previous 12 months, as determined using healthcare utilisation data from the household survey. At the visit, it was assumed that healthcare assistants inform the patient of their eligibility to be screened and invite them to be tested for AF. A public health officer then uses AliveCor to screen all eligible and willing patients. Patients with a positive, inconclusive or unreadable AliveCor result are assessed by a primary healthcare doctor and a 12-lead ECG is performed when available. Patients with a positive AliveCor result, in addition to 10% of patients with an inconclusive or unreadable AliveCor result, are referred to a cardiologist. The cardiologist reviews the AliveCor results and orders a 12-lead ECG for all patients referred. We compared systematic and targeted screening with the opportunistic screening and considered opportunistic screening as ‘usual care’.

The following assumptions were made within these model structures:

1. AliveCor test was done at a single time point.
2. Death was not considered in the model given assumption 1.
3. A smart mobile device and a laptop were available in every primary healthcare institution.
4. Both systematic and targeted screening strategies were to be conducted by a public health officer at a primary healthcare setting (using AliveCor).
5. A 12-lead ECG was available for 20% of screened individuals in a primary healthcare facility.
6. A 12-lead ECG was available for 100% of individuals that were referred to a tertiary hospital.

### Model parameters

#### Incidence data (screening outcome)

The main measure of effectiveness was the number of new detected AF cases by each screening strategy. Data on the number of incident AF diagnoses were calculated from the cross-sectional study: only new AF diagnoses were considered, that is, patients with a history of AF were excluded from the calculation. For the systematic screening, this included all new AF diagnoses, whereas for the targeted screening, only new AF diagnoses among those aged 60 years or older with one of the aforementioned risk factors were considered, and for the opportunistic screening, only new AF diagnoses from those that had an eligible healthcare visit within the previous 12 months were considered.

#### Resource use and costs

Healthcare resources associated with screening, including staff time and use of AliveCor and 12-lead ECGs, were determined based on international guidelines^9^ and discussion with experts. Equipment and staff costs^25^ (monthly salary and staff time needed for screening) used for the screening strategies were calculated based on expert knowledge, local clinicians (MG and SK) and national published data.^25^,^30^ Staff time was calculated to include time required to identify eligible individuals (targeted and opportunistic screening), invite individuals via phone (systematic and targeted screening), explain the test to eligible individuals (opportunistic screening) and verification of all AliveCor results by a primary healthcare doctor in addition to a 12-lead ECG examination by an ECG technician where one is available in primary care. Time required for a cardiologist in a tertiary hospital to review and confirm the results and initiate AF care was also considered. Staff time was multiplied by staff salary (per minute) to generate the total cost of staff time. Detailed information on resource utilisation is described in table 1.

**Table 1:** Resource use details and assumptions

Resource use	Unit per participant	Description/assumptions	Applicability to screening strategy
Staff time			
Public health officer	5 min	Provide information to the participant+conduct AliveCor test	Opportunistic
Public health officer	40 min	Travel time	Systematic and Targeted—considered in the subgroup analysis
Healthcare assistant			
Flagging—opportunistic	2 min	Flagging potential participants who are visiting healthcare settings	Opportunistic
Flagging—systematic	1 min	Identify participants	Systematic
Flagging—targeted	2 min	Identify high risk participants	Targeted
Invitation	2 min	Call to invite	Systematic and targeted
Book appointments	1 min	–	Systematic and targeted
In-person explanation	3 min	Explain option of performing the AliveCor ECG during current visit	Opportunistic
Primary healthcare doctor	5 min	Review AF cases (only 5% of inconclusive and unreadable cases)	All
Cardiologist	10 min	Confirm AF cases (including AliveCor AF+ve, inconclusive and unreadable cases)	All
12-lead ECG examination			
ECG technician	10 min	Perform ECG	All
Cardiologist	1 min	Interpret ECG result	All
ECG electrode gel	5 mL	Amount per visit	All
ECG electrodes	10	6 chest+4 limb leads	All
Other resources for model and scenarios			
AliveCor App and device		14 devices for 10 000 participants	All
Mobile device		14 units for 10 000 participants	All
Laptop		4 units for 10 000 participants	All
Phone calls/SMS		Strategy specific (applied to invite and referral)	All
Travel by car		10 km per visit	Systematic and targeted

AF, atrial fibrillation.

The cost of performing the AliveCor test only comprised the cost of purchasing the AliveCor device. The cost of a 12-lead ECG was also considered and added to the cost of screening.^31^ All equipment costs were annuitised over a 3-year period (estimated average lifespan of equipment), and discounted at 5% which is recommended for estimating future costs in LMICs.^32^ All costs are initially reported in Sri Lankan rupees (LKR) based on 2022 rates. Costs are also reported in US dollars using the 2022 exchange rate of US$1=Rs 329. Information on resource unit cost values/prices is presented in table 2. The total cost of screening was calculated for the three different screening strategies. The annual cost of managing strokes was obtained from the literature^33^ (table 2).

**Table 2:** Resource unit costs in Sri Lankan rupees for 2022

Resource item	Unit cost	Description	Source
Staff salary per hour			
Public health officer	Rs 510	Basic monthly wage of Rs 88 700—173 working hours per month	26
Cardiologist	Rs 1960	Basic monthly wage of Rs 340 000—173 working hours per month	25
Primary care doctor	Rs 1220	Basic monthly wage of Rs 211 000—173 working hours per month	27
Assistant/health attendant	Rs 280	Basic monthly wage of Rs 48 100—172 working hours per month	28
ECG technician	Rs 192	Basic monthly wage of Rs 32 970—172 working hours per month	29
Nurse	Rs 420	Basic monthly wage of Rs 73 500—175 working hours per month	30
12-lead ECG examination			
12-lead ECG—tertiary hospital	Rs 500 000	Serving 12 000 patients per year	Expert consultation
12-lead ECG—primary healthcare setting	Rs 500 000	Serving 520 patients per year	Expert consultation
ECG electrodes	Rs 15 000	10 electrodes (used by 100 patients) per year	Expert consultation
ECG gel	Rs 690	260 mL gel	Expert consultation
Other resources for model and scenarios			
AliveCor	Rs 19 800		44
Laptop	Rs 165 000		Local consultation
Mobile device	Rs 30 500		Local consultation
Phone calls	Rs 2	Price per minute	45
Transportation—car	Rs 300	Per 1 km	Local consultation
CVD management—annual cost			
Stroke management	Rs 4 843 336		33
Warfarin	Rs 10 178	Initial dose: 5–10 mg daily for 4 weeks Maintenance dose: 3–9 mg daily	Expert consultation
INR test	Rs 15 000	Average of 12 tests per year	Expert consultation

CVD, cardiovascular disease; INR, international normalised ratio; Rs, Sri Lankan rupees.

#### Stroke extrapolation

We determined the cost of treating newly detected AF cases for preventing stroke to evaluate the consequences of early detection of AF for the first year after detection. The number of anticipated stroke cases for the different screening strategies was estimated. Evidence from LMICs suggests that 40% of diagnosed patients are likely to receive AF management.^6^ The annual risk of developing stroke for patients with AF treated with warfarin is 3.05%^21^ and this is likely to increase to 9.5%^34^ if those patients were not treated. These estimates were used to calculate the number of incident stroke episodes within a year in patients with diagnosed AF in each screening strategy and to determine the proportion of prevented stroke calculated from the number of invited individuals. The costs associated with AF care and managing strokes were added to the cost of AF diagnosis for each strategy.

### Analysis

Microsoft Excel (V.2023) was used to create the model that featured an easily customisable user interface where users were allowed to choose from a variety of scenarios to produce model outputs. The model also allows users to change the number of screened individuals with the possibility of varying the level of availability of a 12-lead ECG examination in primary healthcare settings following an AliveCor test.

For comparing the three strategies (base case analysis), the analysis followed the care pathways and model inputs as previously described. A subgroup analysis was conducted on all three strategies to evaluate the cost-effectiveness if only individuals over the age of 60, 70 or 80 years were included. Three scenarios were explored to evaluate the cost-effectiveness of the three screening strategies using different assumptions from the base case analysis. These scenarios included (1) cost of public health officers travelling to individuals’ homes for screening, (2) nurses performing the AliveCor and (3) the cost for purchasing mobile devices and laptops for primary care. Finally, analysis was undertaken to consider a hypothetical ‘usual care’ with opportunistic detection of cases but at 50% of the cost and cases detected of the opportunistic screening strategy.

An additional analysis was conducted to evaluate the cost-effectiveness of early AF detection on stroke outcomes. It was envisaged that 40% of patients diagnosed with AF receive ongoing care for 1 year involving monthly visits with a cardiologist at the cardiology clinic and appropriate treatment including treatment with warfarin and regular monthly visits for international normalised ratio test to monitor blood thinning. The cost per percentage of stroke avoided was calculated by dividing the incremental total cost by the incremental proportion of prevented stroke episodes calculated for the three strategies.

### Patient and public involvement

Two people with AF aided in the conceptualisation, planning and delivery of the cross-sectional survey.

## Results

The cross-sectional study included 10 000 participants, 53 (0.5%) of which had a history of AF, 9938 (99.4%) took the AliveCor test and 48 (0.5%) were found to have a new diagnosis of AF; this data was used for calculating costs for systematic screening. Data collected from the cross-sectional study were used to determine the following figures for targeted and opportunistic screening, respectively: 7780 and 6556 people would participate; 52 (0.7%) and 48 (0.7%) would have a history of AF, 7724 (99.3%) and 6502 (99.2%) would take the AliveCor test; 47 (0.6%) and 30 (0.5%) would be newly diagnosed with AF.

### Base case and subgroup analysis

The results of the economic model, including the number of newly detected AF cases and the associated total cost of each screening strategy are presented in table 3. Systematic screening was more expensive with a total cost of Rs 698 422 (US$2123) compared with Rs 492 002 (US$1496) for the targeted screening. Opportunistic screening was cheaper with a total cost of Rs 360 617 (US$1096); however, this strategy only identified 30 new AF cases. The results are also shown in a cost-effectiveness frontier (online supplemental figure 1).

**Table 3:** Results of the cost-effectiveness analysis—base case analysis in Rs and US$ based on 2022 rates

	Total aggregated cost in LKR (US$)	New detected AF cases	Average cost per case detected	Incremental costs	Incremental case detected	ICER (cost per case detected)
Strategy						
Opportunistic screening n=6556	Rs 360 617 (US$1096)	30	Rs 12 021 (US$37)	–	–	–
Targeted screening n=7780	Rs 492 002 (US$1496)	47	Rs 10 468 (US$32)	Rs 131 385 (US$399)	17	Rs 7729 (US$23)
Systematic screening n=10 000	Rs 698 422 (US$2123)	48	Rs 14 550 (US$44)	Rs 206 420 (US$628)	1	Rs 206 420 (US$628)
Additional comparisons						
Systematic vs opportunistic	–	–	–	Rs 337 805 (US$1027)	18	Rs 18 767 (US$57)

AF, atrial fibrillation; ICER, incremental cost-effectiveness ratio; n, number of participants; Rs/LKR, Sri Lankan rupees.

The cost of staff time was the main cost component and contributed to over 55% of the total cost for each screening strategy. The results of the cost-effectiveness analysis suggest that targeted screening is likely to be the most cost-effective strategy when compared with opportunistic screening with an Rs 7729 (US$23) per additional detected AF case. The ICER of systematic screening compared with opportunistic screening was higher at Rs 18 767 (US$57) per detected AF case. When the systematic screening strategy was compared with targeted screening, the cost per additional detected AF case increased to Rs 206 420 (US$628).

The subgroup analysis (online supplemental table 1) suggested that there was no difference in effect between targeted and systematic screening, across patient groups over the age of 60; however, targeted screening costs were higher due to more administration time for identifying eligible patients. Compared with both targeted and systematic screening, opportunistic screening remained less costly but considerably less effective in identifying new AF cases.

### Scenario analyses

When travel expenses were included for public health officers visiting participants’ homes (rather than screening undertaken in a primary care setting), the total cost of systematic and targeted screening increased substantially to Rs 6 085 360 (US$18 497) for 10 000 screened individuals and Rs 3 749 027 (US$11 395) for 7780 screened individuals, respectively (online supplemental table 2). Travel expenses accounted for over 85% of the total cost and therefore this approach was not efficient. The cost of opportunistic screening remained unchanged as no travel was required. The costs were reduced by 11% for systematic screening and 9% for targeted and opportunistic screening in the scenario where nurses perform the AliveCor instead of a public health officer. In the scenario where mobile devices and laptops are purchased for primary care units, the costs were increased by 57% for systematic, 49% for targeted and 47% for opportunistic screening. Finally, when comparing hypothetical ‘usual care’ (with 50% of costs and cases detected) with opportunistic screening, the ICER was Rs 12 021 (US$37) per case detected and targeted screening remained the most cost-effective option (online supplemental table 3).

### Stroke extrapolation

Based on 12 months of care provided for newly detected AF patients, 21 600 individuals need to be screened using the systematic screening strategy to prevent one stroke episode for Rs 35 498 494 (US$107 915) (online supplemental table 4). Similarly, 17 272 individuals need to be screened in the targeted screening to prevent one stroke with an associated cost of Rs 36 171 872 (US$109 962). For the opportunistic screening strategy, 22 684 individuals would need to be screened to prevent one stroke at a cost of Rs 57 759 084 (US$175 588).

## Discussion

In this economic evaluation of AF screening in Sri Lanka, our principal findings are as follows: (1) systematic screening and targeted screening detected similar numbers of new AF cases, but systematic screening was the most expensive strategy in our main analysis of individuals aged ≥50 years; (2) opportunistic screening was the least costly but considerably less effective in identifying new AF cases; (3) when restricted to individuals aged ≥60 years, targeted screening would cost more than systematic screening, and both would be equally effective in identifying new AF cases; and (4) targeted screening was calculated as the most effective strategy in terms of preventing stroke episodes.

We found that screening for AF using a handheld ECG device (AliveCor) among those aged 50 years or older with hypertension, diabetes or cardiovascular disease (ie, targeted screening) was the most cost-effective approach in our setting where there is currently no existing screening programme. When restricting the analysis to individuals aged 60 years or older, systematic screening was more cost-effective; however, systematic screening identified four fewer AF cases and targeted screening identified five fewer AF cases when compared with using an age threshold of ≥50 years. Using AliveCor has the potential to provide added benefit of early AF detection without adding much burden on primary care staff (10 min per patient for targeted screening). Preliminary findings from an ongoing qualitative project with primary care staff in Sri Lanka indicate that incorporating AliveCor into routine practice to screen those that meet a particular criterion (ie, targeted screening) would be feasible and acceptable given the appropriate training is provided. Future research should assess the real-world feasibility and effectiveness of incorporating AliveCor into routine care. However, existing programmes such as opportunistic pulse palpation conducted in the community^34^ have successfully been complemented with handheld devices such as AliveCor.^35^ Therefore, adding the AliveCor onto an existing programme elsewhere may reduce the overall costs for a systematic or opportunistic strategy.

While early detection of AF can result in life-saving treatment, the subsequent management of newly detected AF cases needs an appropriate care pathway. An effective pathway should focus on avoiding stroke, better symptom management with patient-centred rate or rhythm control, and the management of comorbidities and lifestyle changes, referred to as Atrial fibrillation Better Care (ABC) pathway.^36^ Adherence to the ABC pathway has been associated with improved clinical outcomes and healthcare cost savings^37 38^ and recommended in guidelines, including those from the Asia-Pacific region.^39 40^ The present study shows how targeted AF screening can detect new AF cases to ‘feed’ into the care pathway. However, once AF detection increases, understanding how to help people with AF cope with side effects and treatment is crucial given the potential decline in quality of life.^41^ Indeed, a separate study of ours showed that people with AF in Sri Lanka often compromise on routine and important activities such as attending family events and funerals.^13^

This study has a major strength in using available data from a large community-based cross-sectional study involving 10 000 individuals, allowing for three different strategies to be compared. Existing evidence regarding the cost-effectiveness of AF screening has been conducted in high-income countries, incorporating sophisticated monitoring approaches or biomarkers^42 43^; however, this is the first study to be conducted in any LMIC.

However, there are some limitations to mention. Usual care is difficult to define in Sri Lanka as AF is typically picked up opportunistically. We chose opportunistic screening as the usual care comparator; however, this may be an overestimate in terms of costs and cases detected. Therefore, we undertook a sensitivity analysis where usual care was halfway (in terms of costs and cases detected) between ‘do nothing’ (no costs and no cases detected) and opportunistic screening, and targeted screening remained cost-effective. While we used ‘AF cases detected’ as the primary outcome due to data availability, future research should undertake Markov modelling over a longer time horizon to incorporate disability-adjusted life years averted or quality-adjusted life years gained from stroke prevention to align with global cost-effectiveness standards. The lack of sensitivity analyses is a limitation of this study since only mean values from secondary data sources were used without ranges or measures of uncertainty. This limited our ability to formally assess robustness. In future research, incorporating ranges from available evidence or applying reasonable variations to key parameters could help better capture and explore uncertainty. Another limitation to mention is the use of cost estimates for stroke management from a 2014 Indian study in the absence of Sri Lanka-specific data. These costs, which included stroke-related hospitalisation and outpatient visits, are considered the most relevant data and were adjusted to prices using Sri Lanka’s inflation rate. However, the economic crisis that was occurring at the time of this study may have resulted in relatively high estimates, underlining the need for local data. Finally, phone-based screening assumes universal mobile access, which may underestimate the effectiveness of targeted screening; however, home visits (explored in our sensitivity analysis) remain logistically challenging and not cost-effective.

Compared with opportunistic screening, targeted screening with AliveCor was the most cost-effective strategy (Rs 7729 per additional case detected), balancing yield and costs. Systematic screening, while having similar effectiveness, was not cost-effective due to the high additional costs to detect just one further case. These findings support integrating targeted screening into Sri Lanka’s primary care pathways. With better detection of AF, more people can receive timely and effective medications to reduce morbidity and premature mortality in Sri Lanka and other LMICs where the burden of AF is expected to increase as the life expectancy continues to increase.

## Supplementary material

10.1136/bmjgh-2025-019592 online supplemental file 1

10.1136/bmjgh-2025-019592 online supplemental figure 1

## Data Availability

Data are available upon reasonable request.
