# Supplementary material for: Cost-effectiveness of three screening strategies for atrial fibrillation in Sri Lanka: a decision-tree modelling analysis using community-based prevalence data
Source: BMJ Glob Health. 2026 Mar 13;11(3):e019592. doi: 10.1136/bmjgh-2025-019592 (PMC12993347; doi:10.1136/bmjgh-2025-019592)
Supplement: online supplemental file 1 [file bmjgh-11-3-s001.docx]

**Supplementary materials**

Table 1. Sub-group analysis in Sri Lankan Rupees (Rs) and USD ($) based on 2022 rates

| **Strategy** | **Total aggregated cost in LKR (USD)** | **New detected AF cases** | **Average cost per case detected** | **Incremental costs**  **LKR (USD)** | **Incremental cases detected** | **ICER (cost per case detected)**  **LKR (USD)** |
| --- | --- | --- | --- | --- | --- | --- |
| **Age 60 years or above** |  |  |  |  |  |  |
| Opportunistic Screening n= 4,528 | Rs. 207,231 ($630) | 27 | Rs. 7,675 ($23) | - | - | - |
| Systematic Screening  n= 6,746 | Rs. 360,190 ($1,095) | 43 | Rs. 8,377 ($25) | Rs. 152,959 ($465) | 16 | Rs. 9,560 ($29) |
| Targeted Screening  n= 6,746 | Rs. 391,694 ($1,191) | 43 | Rs. 9,109 ($28) | Rs. 31,504 ($96) | 0 | Dominated by systematic screening (more costly with no extra cases detected) |
| **Age 70 years or above** |  |  |  |  |  |  |
| Opportunistic Screening n= 2,069 | Rs. 71,849 ($218) | 18 | Rs. 3,992($12) | - | - | - |
| Systematic Screening  n=3,133 | Rs. 107,312 ($326) | 32 | Rs. 3,354 ($10) | Rs. 35,463 ($108) | 14 | Rs. 2,533 ($8) |
| Targeted Screening  n= 3,133 | Rs. 121,943 ($371) | 32 | Rs. 3,811 ($12) | Rs. 14,631 ($45) | 0 | Dominated by systematic screening (more costly with no extra cases detected) |
| **Age 80 years or above** |  |  |  |  |  |  |
| Opportunistic Screening n= 386 | Rs. 9,856 ($30) | 2 | Rs. 4,928 ($15) | - | - | - |
| Systematic Screening  n=636 | Rs. 12,755 ($39) | 7 | Rs. 1,822 ($6) | Rs. 2,899 ($9) | 5 | Rs. 580 ($2) |
| Targeted Screening  n= 636 | Rs. 15,725 ($48) | 7 | Rs. 2,246 ($7) | Rs. 2,970 ($9) | 0 | Dominated by systematic screening (more costly with no extra cases detected) |

Abbreviations: AF, Atrial Fibrillation; ICER, Incremental Cost-Effectiveness Ratio; n, number of participants.

Table 2. Results of the scenario analysis - in Sri Lankan Rupees (Rs) and USD ($) based on 2022 rates

| **Scenario/strategy** | **Total aggregated cost in LKR (USD)** | **New detected AF cases** | **Cost per case detected** | **Incremental costs**  **LKR (USD)** | **Incremental cases detected** | **ICER (cost per case detected)**  **LKR (USD)** |
| --- | --- | --- | --- | --- | --- | --- |
| **Travel to patients’ houses** |  |  |  |  |  |  |
| Opportunistic Screening n= 6,556 | Rs 360,617 ($1,096) | 30 | Rs. 12,021 ($37) | - | - | - |
| Targeted Screening  n= 7,780 | Rs 3,749,027 ($11,395) | 47 | Rs. 79,767 ($242) | Rs. 3,388,410 ($10,299) | 17 | Rs. 199,318 ($606) |
| Systematic Screening  n=10,000 | Rs 6,085,360 ($18,497) | 48 | Rs. 126,778 ($385) | Rs. 2,336,333  ($7,102) | 1 | Rs. 336,333 ($7,102) |
| **AliveCor test performed by a nurse** |  |  |  |  |  |  |
| Opportunistic Screening n= 6,556 | Rs. 327,575 ($996) | 30 | Rs. 10,919 ($33) | - | - | - |
| Targeted Screening  n= 7,780 | Rs. 445,430 ($1,354) | 47 | Rs. 9,477 ($29) | Rs. 117,855 ($358) | 17 | Rs. 6,933 ($21) |
| Systematic Screening  n=10,000 | Rs. 621,395 ($1,889) | 48 | Rs. 12,946 ($39) | Rs. 175,965 ($535) | 1 | Rs. 175,965 ($535) |
| **Purchase of mobile phones and computers** |  |  |  |  |  |  |
| Opportunistic Screening n= 6,556 | Rs. 530,791 ($1,614) | 30 | Rs. 17,693 ($54) | - | - | - |
| Targeted Screening  n= 7,780 | Rs. 731,865 ($2,225) | 47 | Rs. 15,572 ($47) | Rs. 201,074 ($611) | 17 | Rs. 11,828 ($36) |
| Systematic Screening  n=10,000 | Rs. 1,095,143 ($3,329) | 48 | Rs. 22,815 ($69) | Rs. 363,278 ($1,104) | 1 | Rs. 363,278 ($1,104) |

Abbreviations: AF, Atrial Fibrillation; ICER, Incremental Cost-Effectiveness Ratio; n, number of participants.

Table 3. Results of the cost -effectiveness analysis with hypothetical ‘usual care’ strategy - in Sri Lankan Rupees (Rs) and USD ($) based on 2022 rates

| **Strategy** | **Total aggregated cost in LKR (USD)** | **New detected AF cases** | **Average cost per case detected** | **Incremental costs**  **LKR (USD)** | **Incremental cases detected** | **ICER (cost per case detected)**  **LKR (USD)** |
| --- | --- | --- | --- | --- | --- | --- |
| ‘Usual care’ opportunistic detection | Rs. 180,309 ($548) | 15 | Rs. 12,021 ($37) | - | - | - |
| Opportunistic Screening n= 6,556 | Rs 360,617 ($1,096) | 30 | Rs 12,021 ($37) | Rs. 180,309 ($548) | 15 | Rs. 12,021 ($37) |
| Targeted Screening  n= 7,780 | Rs 492,002 ($1,496) | 47 | Rs 10,468 ($32) | Rs. 131,385 ($399) | 17 | Rs. 7,729 ($23) |
| Systematic Screening  n=10,000 | Rs 698,422 ($2,123) | 48 | Rs 14,550 ($44) | Rs. 206,420 ($628) | 1 | Rs.206,420 ($628) |

Abbreviations: AF, Atrial Fibrillation; ICER, Incremental Cost-Effectiveness Ratio; n, number of participants.

Table 4. Cost of AF treatment and stroke management to prevent one stroke episode per strategy in Sri Lanka

| **Strategy** | **Total cost in LKR (USD)*** | **Number of all individuals** | **Number of individuals screened** | **Proportion (number of individuals screened/ total population)** |
| --- | --- | --- | --- | --- |
| Systematic Screening | Rs. 35,498,494 ($107,915) | 21,600 | 21,600 | 100% |
| Targeted Screening | Rs 36,171,872  ($109,962) | 22,200 | 17,272 | 78% |
| Opportunistic Screening | Rs 57,759,084 ($175,588). | 34,600 | 22,684 | 66% |

Abbreviations: AF, Atrial Fibrillation; ICER, Incremental Cost-Effectiveness Ratio; LKR, Sri Lanka Rupee; USD, United States Dollar.

*Cost includes the cost of AF diagnosis, treating 40% of new AF cases and managing stroke episodes.
