# Supplementary figures and images for: Cost-effectiveness of three screening strategies for atrial fibrillation in Sri Lanka: a decision-tree modelling analysis using community-based prevalence data
Source: BMJ Glob Health. 2026 Mar 13;11(3):e019592. doi: 10.1136/bmjgh-2025-019592 (PMC12993347; doi:10.1136/bmjgh-2025-019592)

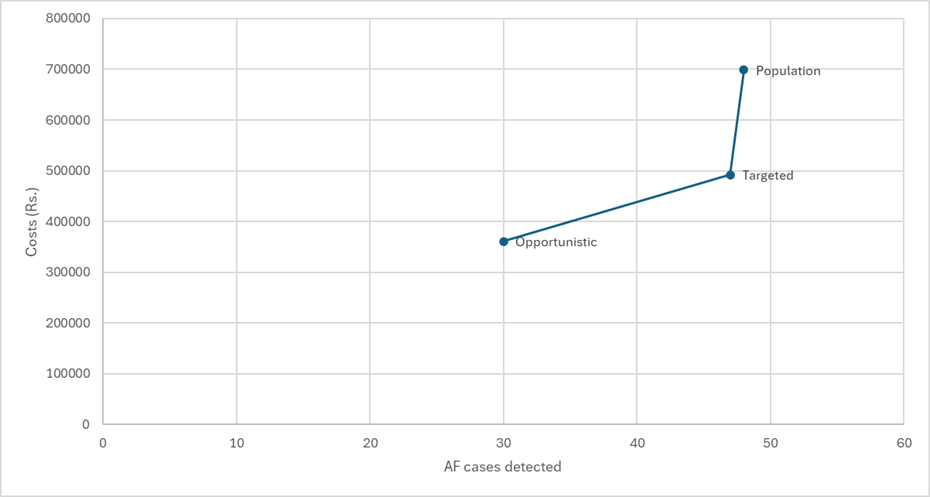

Supplement: online supplemental figure 1 [file bmjgh-11-3-s002.tif]
